# Research on the mechanism of information infrastructure affecting industrial structure upgrading

**DOI:** 10.1038/s41598-022-24507-9

**Published:** 2022-11-19

**Authors:** Pei Zhang, Jiaoe Wang, Mengming Li, Fan Xiao

**Affiliations:** 1 Key Laboratory of Regional Sustainable Development Modeling, Institute of Geographic Sciences and Natural Resources Research, China Academy of Sciences, Beijing, China; 2 College of Resources and Environment, University of Chinese Academy of Sciences, Beijing, China; 3 Food and Resource Economics Department, University of Florida, Gainesville, FL USA; 4 Faculty of Sciences, DGES-IGEAT, Université Libre de Bruxelles (ULB), 1050 Brussels, Belgium

**Keywords:** Environmental social sciences, Socioeconomic scenarios, Sustainability

## Abstract

The construction of information infrastructure as well as the transformation and upgrading of the industrial structure are among the major challenges for the Chinese economy. Therefore, it is of great significance to explore how information infrastructure affects the upgrading of industrial structure. Based on the panel data of 31 provinces in China from 2013 to 2020, mediating effect model and non-parametric percentile bootstrap method are used to carry out empirical research, by creating an information infrastructure construction level and industrial structure upgrading indicators. The results show that, in addition to the direct effect of information infrastructure on industrial structure upgrading, information infrastructure can also work indirectly through three paths: (1) information infrastructure acts on industrial structure upgrading by enhancing urbanization level; (2) information infrastructure affects industrial structure upgrading by boosting technological innovation; (3) information infrastructure first enhances urbanization level, then acts on technological innovation, and finally promotes industrial structure upgrading. In addition, the intermediary effect of technological innovation is stronger than urbanization. In general, this study acknowledges that urbanization and technological innovation are partial mediators in the process of information infrastructure affecting industrial structure upgrading, notwithstanding other potential impact pathways being studied further.

## Introduction

With the deepening and fastening pace of a new round of global technological revolution and industrial reform, information infrastructure plays an increasingly important role in industrial reform. In December 2018, a new concept of New Infrastructure was proposed for the first time at the Central Economic Work Conference in China. Afterward, information infrastructure such as Internet of Things (IoT), artificial intelligence (AI), blockchain, and their applications have gradually penetrated almost all areas of the economy and society. At the same time, the economy in China is undergoing a transformation from high-speed development to high-quality development^1^, and industrial structure upgrading is said to play a key role in this transformation^2^.

However, the relationship between information infrastructure and industrial structure upgrading is still unclear, which some scholars even regard as an assemblage of urbanization and technology^3–6^.Can information infrastructure play a role in transforming and upgrading the industrial structure? What is the transmission mechanism of this effect? Our research is about to explore and answer some of those critical questions. Specifically, this paper will focus on China’s information infrastructure and explore its impacts on industrial structure upgrading in terms of direct and indirect impact pathways. According to studies by Kuznets, the basic law of economic structure transformation implies that the ratio of value-added in the primary industry to total GDP is decreasing, while those ratios of value-added in the secondary and tertiary industries, especially the service industry, is increasing^7^. The past economic development in China conforms to those abovementioned facts^8^. Based on those facts, this paper views the declines in the proportion of the primary industry and rises in the proportion of the secondary and tertiary industries as the signals of industrial structure upgrading and accordingly constructs the industrial structure index.

Given the costliness of information infrastructure construction and the urgency of China’s industrial transformation and upgrading, an in-depth analysis of the relationship between the two and its underlying mechanism is of great significance to optimize resource allocation in space and promote China’s sustainable economic development. Many scholars have analyzed the impact of information infrastructure on industrial structure upgrading, and these studies mainly focus on the following two aspects. On the one hand, some scholars have explored the impact of information infrastructure on industrial structural upgrading based on the perspective of a particular type of information infrastructure or the technology it provides. For example, Zhang et al.^9^ find that broadband infrastructure improves firm productivity through mechanisms such as information exploitation, redistributive efficiency, improved innovation efficiency, and reduced transaction costs, which in turn drive industrial structural upgrading; Acemoglu and Restrepo^10^ argue that artificial intelligence while improving workers’ skills and stimulating technological innovation, promotes industrial structural transformation and has an impact on allocative efficiency and exchange patterns. In addition, information infrastructure will promote the construction of information sharing platforms as a way to grasp demand-side consumption trends and guide industrial structural upgrading^11^, as well as influence industrial structural upgrading by providing information technology to facilitate service outsourcing^12^. On the other hand, some scholars have found that by influencing industrial structure upgrading, information infrastructure will then indirectly affect other areas, such as economic growth^13^, air pollution^6,14^, eco-efficiency^15^, energy consumption^16^, and green total factor productivity^17^. In summary, the established studies have accumulated rich experience for the research on the influence of information infrastructure on industrial structure upgrading. However, there are still the following points that can be improved. First, information infrastructure is a complex and comprehensive system, and its comprehensive scientific evaluation needs to be further strengthened. second, the path of information infrastructure influencing industrial structure upgrading still needs to be further explored.

Against this background, this paper will evaluate comprehensively evaluate information infrastructure, and then reveal what is the mediating path for the information infrastructure to influence industrial structure upgrading. Among them, this paper evaluates information infrastructure comprehensively by the entropy-TOPSIS method, which can not only avoid the influence of subjective factors to determine the evaluation index but also use the way of approaching the ideal solution to determine the ranking of evaluation objects^18–20^. On this basis, drawing on the studies of Wen^21^ and Zhang et al.^6^, it is appropriate to adopt the mediating effect to explore the independent variable X to influence the dependent variable Y through the influence variable M model. Thus, this paper will use the mediating effect model to explore the mediating path of information infrastructure affecting industrial structure upgrading.

The major contributions of this paper are reflected in the following two aspects: (1) to build a theoretical model that depicts how information infrastructure impacts industrial structure upgrading; (2) to specifically evaluate and analyze the impacts mechanisms and pathways of information infrastructure on industrial structure upgrading. The rest of this paper is arranged as follows: the second part discusses the impact mechanisms of information infrastructure on industrial structure upgrading, and constructs theoretical models and research assumptions; the third part introduces the setting of empirical models, data sources, and variables selection; the fourth part examines the impacts of information infrastructure on the industrial structure upgrading and robustness tests, to reveal that the impact mechanisms and pathways of information infrastructure on industrial structure upgrading; the last parts conclude this paper.

## Conceptual framework and hypothesis development

### Information infrastructure and industrial structure upgrading

The impacts of information infrastructure have obvious spillover characteristics, which provide the general technologies to promote the fourth industrial revolution^22,23^ so that the impacts can comprehensively affect all industries of the economy. On one hand, information technology innovation brought by improving information infrastructure has continuously spawned new industries, new formats, and new modes, and the emergence of new industries has further accelerated the optimization and upgrading of industrial structure. On the other hand, information technology enables traditional industries to realize the transformation and upgrading to promote industrial structure upgrading. In essence, constructing information infrastructure will allow regions to develop industries according to their comparative advantages, and then achieve growth through trade. The specialization of labor division and convenience of the transaction will promote industrial agglomeration and continuously accumulate favorable factors, thus forming a “growth pole” in self-strengthening. At the same time, industrial agglomeration and division of labor brought about by information infrastructure may also bring the “diffusion effect”, that is, regions with the relatively less advanced industrial structure can take advantage of their comparative advantages to undertake some industries from other advanced regions, or further strengthen the development of advantageous industries through knowledge spillover and technical exchange. Exogenous comparative advantage, endogenous specialized economy, and transaction efficiency determine the division of labor and trade patterns among regions^24,25^, while information infrastructure improves transaction efficiency and accelerates the division of labor and specialization among regions. In addition, the construction of information infrastructure will further promote regional economic integration, accelerate the flow of factors, and increase enterprise cooperation. Active learning activities among enterprises, such as learning the advanced production technologies from other enterprises through technical personnel exchange, on-the-spot investigation, patent citation, etc^26,27^, promote the improvement of production technologies and the optimization of factor input structure of enterprises, hence, to realize the transformation and upgrading of the overall industry.

## Hypothesis 1: construction of information infrastructure induces industrial structure upgrading

Furthermore, in addition to direct effects, the Engel effect and the Baumol effect are driven by information infrastructure and are likely to have impacts on the transformation and upgrading of China’s industrial structure. Among them, the Engel effect is derived from Engel’s law, which emphasizes the impacts of product demand income elasticity in different industrial sectors. As the income elasticity of demand for agricultural products is lower than that of non-agricultural products, the increase in income will make the demand for non-agricultural products rise faster, which will drive the transfer of labor force to non-agricultural industries^28^, thus accelerating the pace of urbanization^29,30^. Although artificial intelligence brought about by the construction of information infrastructure may also replace labor^10^, information infrastructure promotes automated production and only replaces a part of labor. On the contrary, information infrastructure will increase the demand for specific labor that is difficult to be automated and requires strong information skills and eventually might not reduce the overall labor demand^22,23,31,32^. Acemoglu and Restrepo^32^ estimated that the application of automation might make 75million to 375million people change their jobs. Therefore, the construction of information infrastructure may accelerate the process of urbanization. At the same time, the Engel effect is often introduced by setting consumers with hierarchic preferences, which are endogenous. Because urban cities have more consumer preferences and types of consumer goods compared to consumers in rural areas^33,34^, this will greatly impact the transformation and the upgrading of industrial structure. Therefore, information infrastructure will drive industrial structure upgrading by promoting the process of urbanization.

## Hypothesis 2: the construction of information infrastructure promotes industrial structure upgrading by accelerating urbanization

The Baumol effect was put forward by Baumol^35^. It expounds that output growth is positively correlated with labor productivity growth and emphasizes that the difference in labor productivity in various industries will affect the relative prices of products. Suppose the products of different industrial sectors are complementary to each other. In that case, the product prices of industrial sectors with rapid technological innovation are relatively low, which will promote the transfer of labor to these industrial sectors^36^, thus promoting the industrial structure upgrading. Technological innovation or progress has played an important role in industrial structure upgrading. However, information infrastructure is an important impetus for technological innovation because its construction has laid the foundation for the widespread application of IT technology. By building an intelligent network technology platform, the speed of super large-scale information transmission, the sharing of the latest R&D achievements, and the spillover of cutting-edge knowledge and technology have been greatly improved. Also, the cost of network search and information transmission has been reduced. At the same time, the dissemination and exchange of information and knowledge among different entities have been accelerated^37,38^. Hence, the marginal returns of technology have been increased through the spillovers of knowledge and technology, enhancing technological innovation’s compatibility and scalability. At the same time, the construction of information infrastructure can effectively break the time and space constraints of new information exchange, promote the transfer and flow of innovation elements and intellectual capital among regions and industries^39^, and enhance R&D cooperation and technical exchange among enterprises, which will help reduce the risk of innovation uncertainty caused by information asymmetry. Therefore, constructing information infrastructure will promote industrial structure upgrading by fostering scientific and technological innovation.

## Hypothesis 3: the construction of information infrastructure promotes industrial structure upgrading by promoting scientific and technological innovation

### Urbanization and technological innovation

The advantages of urbanization in specialization, diversity, human capital accumulation, information exchange network formation, transaction efficiency improvement, and public services make it conducive to the generation and diffusion of technological innovation. From the perspective of investment in capital and personnel, regional investments in innovation are mainly concentrated in urban areas, and the larger the urban size, the more obvious the advantages of innovation investment in urban areas. Therefore, both the increase in the number of urban cities and the expansion of the urban size will lead to the expansion of the innovation investment scale, which is conducive to the improvement of technological innovation ability. From the perspective of the environment affecting technological innovation, even though the urban and rural areas share the same systems, policies, and laws in the same region, the advantages of urban areas in specialization and diversity, accumulation of human capital, formation of information exchange network and improvement of transaction efficiency make technological innovation still easier to occur in urban areas.

## Hypothesis 4: urbanization promotes technological innovation

Meanwhile, urban areas are concentrated with a larger proportion of the population with different interests, abilities, needs, and wealth, meeting the needs of specialization; A variety of highly specialized enterprises come together to share specific labor markets, information, internal links, and other resources. Different people, enterprises, industries, etc. gathered in urban areas form a diverse environment, create opportunities for exchanges among people in different industries and disciplines, and promote the generation of new knowledge, technology, and industries. Education opportunities and educational infrastructure in urban areas are better than those in rural areas and more conducive to people’s development of their skills, to promote the formation and accumulation of human capital. In big urban cities, innovation practitioners are spatially closer to each other, which is conducive to shortening the distance between each other to enhance mutual trust, reduce the cost of opportunism, and realize communication and cooperation. By gathering the population and industries together, forming stable cooperative relations, and reducing uncertainties, urban areas are conducive to the vertical decomposition of enterprises and promote professional development. The large-scale and well-informed markets in urban areas can develop a series of formal systems to reduce transaction costs, which is conducive to the improvement of transaction efficiency.

In addition, more developed information, communication, and other infrastructure in urban areas provide a good communication channel for innovation diffusion^40^. As arenas of economic activities of high density, urban areas are full of various advertisements, commodity exhibitions, promotional activities, and various academic and commercial conferences, which provide enterprises and individuals with low-cost or even free market and technical information. Moreover, the centralized enterprises and personnel in urban areas have more face-to-face communication opportunities compared to the scattered enterprises and personnel, which makes information spread more effectively. Therefore, urban areas are not only efficient producers of innovation but also provide a favorable environment for innovation diffusion, so that innovation can quickly spread from one person and enterprise to another.

## Hypothesis 5: The construction of information infrastructure promotes industrial structure upgrading, by accelerating urbanization and technological innovation

**Figure 1:**
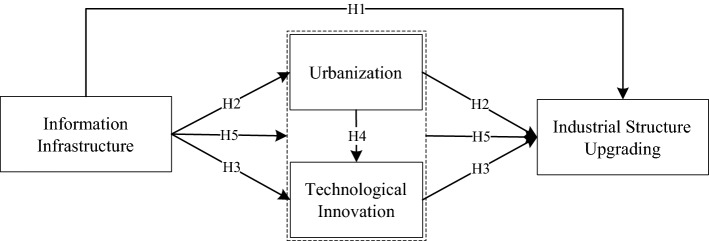
Conceptual framework of the impact of information infrastructure on industrial structure upgrading.

To sum up, we have constructed a theoretical framework (see Fig. 1), which presents several paths for the information infrastructure to affect the upgrading of the industrial structure.

## Methodology and data

### Model specification

Based on the above analysis and assumptions, we will use Baron’s causal steps approach^41^ to evaluate the potential mediating variables involved in the information infrastructure process affecting industrial structure upgrading. Among them, the mediating variables are urbanization and technological innovation according to the hypotheses we proposed above.

An econometric model is built in Eq. (1) to see the impact of information infrastructure on industrial structure upgrading without considering the mediating variables.

1 $${AIS}_{it}=\theta +{\theta }_{1}{INFOR}_{it}+\varphi X+{\gamma }_{t}+{\mu }_{i}+{\varepsilon }_{it},$$

where ${AIS}_{it}$ refers to the industrial structure upgrading index of region $i$ in year $t$, ${INFOR}_{it}$ refers to the information infrastructure level of region $i$ in year $t$, $\theta$ represents the intercept term. $X$ is the group of other control variables that affect industrial structure upgrading, ${\gamma }_{t}$ and ${\mu }_{i}$ denotes the fixed effects of time and areas respectively, and ${\varepsilon }_{it}$ stands for the random error term.

Then, we include the mediating variables in the econometric model, and compare the estimated coefficients, according to the causal steps approach. The exact steps are explained as follows. The first step is to test the impacts of information infrastructure on industrial structure upgrading without mediating variables, as shown in Eq. (2).

2 $${AIS}_{it}={\alpha }_{0}+{\alpha }_{1}{INFOR}_{it}+{\varphi }_{0}X+\varepsilon .$$

The second step is to test the impacts of information infrastructure on mediating variables in Eq. (3):

3 $${MED}_{it}={\beta }_{0}+{\beta }_{1}{INFOR}_{it}+{\varphi }_{1}X+\varepsilon .$$

Step three is to test again the impact of information infrastructure on industrial structure upgrading by controlling the mediating variables.

4 $${AIS}_{it}={\gamma }_{0}+{\gamma }_{1}{INFOR}_{it}+{\gamma }_{2}{MED}_{it}+{\varphi }_{2}X+\varepsilon .$$

As we are discussing two potential mediating variables in this paper, to evaluate the possible interactions between those two variables, that is, whether urbanization affects industrial structure upgrading by influencing technological innovation, additional steps and models are constructed in Eq. (5).

$${TECH}_{it}={\omega }_{0}+{\omega }_{1}{INFOR}_{it}+{\omega }_{2}{UP}_{it}+{\varphi }_{3}X+\varepsilon ,$$

5 $${AIS}_{it}={\omega }_{0}+{\omega }_{1}{INFOR}_{it}+{\omega }_{2}{UP}_{it}+{\varphi }_{3}X+\varepsilon .$$

Variable $Med$ in Eqs. (2) to (5) refers to the mediating variables, and the definitions of other variables are the same as depicted above.

In the analytical framework of the causal steps approach, a significant $\alpha$ is the premise of mediating effect test, that is, it is of practical significance to study the mediating effect only when information infrastructure has a significant impact on industrial structure upgrading. The role of mediating effects can be understood by comparing the significance and the scale of a series of regression coefficients in those models. If ${\beta }_{1}$ and ${\gamma }_{2}$ are significant, and ${\gamma }_{1}$ is insignificant or decreases significantly, there is a mediating effect happening to the process of information infrastructure impacting industrial structure upgrading: when ${\gamma }_{1}$ is insignificant, the mediating variable is a complete mediating variable, however, while ${\gamma }_{1}$ significantly changes before and after the inclusion of mediating variables, there is a partial mediating variable and the possibility of other mediating variables. Similarly, on the basis that we detect urbanization has a significant impact on industrial structure upgrading, if we find the level of urbanization has a significant impact on technological innovation, that is, when ${\omega }_{2}$ is significant and ${\omega }_{1}$ is insignificant or significantly changes, technological innovation plays a mediating effect in the process of urbanization impacting industrial structure upgrading.

Finally, this study also utilizes a nonparametric percentile bootstrap method to further test the mediation paths to consolidate and robust check the causal steps approach. When there are multiple mediators and multi-step mediating effects, the power of the causal steps approach will decrease due to a higher type I error and the false rejection of the existence of mediation effects. Even if the causal steps approach confirms the existence of mediating effects, nonparametric percentile bootstrap tests can also help to verify the results of mediating effect and improve the robustness of the results. Moreover, bootstrap tests can effectively recognize the mediating effects, when there might be multiple mediation variables and complex pathways involved^21^.

## Variables selection

### Dependent variable: industrial structure upgrading

According to Song et al.^42^, we construct the industrial structure upgrading index (*AIS*) of provinces in China as the dependent variable to investigate the mediating effect of information infrastructure on industrial structure upgrading.

6 $${AIS}_{it}=\sum_{n=1}^{3}{Y}_{int}*n,\quad n=1, 2, 3,$$

where, ${Y}_{int}$ refers to the proportion of the total output of the $n$-th industry to the regional GDP of region $i$ in year $t$. AIS index takes values from 1 to 3, which reflects the evolution of the industrial structure from the primary industry to the secondary and tertiary industries. The closer the value is to 3, the higher the level of industrial structure upgrading is.

### Core explanatory variable: information infrastructure

It includes communication network infrastructure represented by 5G, Internet of Things, Industrial Internet, and satellite Internet; new technology infrastructure represented by artificial intelligence, cloud computing, and blockchain; and computing power infrastructure represented by data centers and intelligent computing centers^6,43^. However, whether a single indicator such as cell phone base stations^44^ or fiber optic cable line length^45^ is used, or a composite indicator such as the number of enterprises^6^ or patents^43^ in related fields is used as a proxy variable, it cannot accurately portray the whole picture of information infrastructure. Therefore, this paper combines existing research to select indicators such as cell phone base stations, long-distance fiber optic cable line length, the number of Internet domain names, and Internet broadband access ports (see Table 1) and adopts the entropy-TOPSIS weight method to comprehensively evaluate information infrastructure (*INFOR*).

**Table 1:** Variables used to generate information infrastructure level.

	Description
Information infrastructure	Mobile phone base stations (ten thousand)
	Length of long-distance optical cables (kilometers)
	Number of internet domains (ten thousand)
	Internet broadband access ports (ten thousand)

### Mediating variables

The mediating variables in this study include urbanization (*UP*) and technological innovation (*TECH*). As discussed above, information infrastructure may promote industrial structure upgrading through urbanization and technological innovation. In this study, urbanization level is measured as the proportion of the urban population, and technological innovation is measured as the logarithm value of the number of authorized patents.

### Other control variables

Based on the existing research, this study selects the following control variables to study the impact of information infrastructure on industrial structure upgrading. First, the level of economic development determines the adjustment direction and pace of industrial structure^46,47^, which is also the core reason that economic development affects industrial structure upgrading. Therefore, we use per capita regional GDP to control the level of regional economic development (*PGDP*). Second, regional governance is an important means to make up for market failure and promote the upgrading of industrial structure^48^. Considering that public budget expenditure is the main budget for the regional governance level^49^, this study uses regional per capita public budget expenditure to control the regional governance level (*GOV*). Third, the reallocation of production factors such as human capital is the essence of industrial structure transformation^50^, and the number of college students is a common indicator to measure the level of human capital^51^, hence, we select the proportion of college and university students to control the level of regional human capital (*EDU*). Next, the urban–rural income gap reflects the equality level of the income distribution, and income distribution promotes the transformation and upgrading of the industrial structure by increasing the social demand for domestic goods^52^. Therefore, this study uses the ratio of per capita average disposable income of urban residents to that of rural residents to measure the urban–rural income gap (*INEQ*). Then, population density represents the sizes and scales of both the urban and rural areas. With the expansion of urban areas, mature manufacturing industries that are mostly engaged in standardized production processes will shift to small and medium-sized cities due to low land rent and wages, resulting in the transformation of the industrial structure^53^. Therefore, this study takes the population density (*DPOP*) as the control variable. In terms of the financial environment, financial development promotes economic growth and per capita income by increasing investment and promoting technological innovation, and then consequently affects the transformation of industrial structure from the supply side^54^ or through the “income effect”^28^. Thus, we use the degree of capital deepening to control the level of regional financial development (*FINA*). Last, openness to foreign investment can provide information and direction for industrial structure adjustment. Through foreign trade, regions can be more likely to participate in the international division of labor, absorb relatively mature industries and products from developed regions, and develop their own comparatively advantageous industries, to promote industrial structure upgrading^55^. Therefore, this study uses the logarithm of total import and export volume to control the degree of openness to the outside world (*OPEN*). The descriptive statistical results of the data are as follow (see Table 2).

**Table 2:** The descriptive statistical analysis results of main variables.

Variable	Mean	Std. dev.	Min	Max	No. of observations
*AIS*	2.40	0.12	2.22	2.84	248
*INFOR*	0.18	0.13	0.02	0.72	248
*UP*	0.59	0.13	0.24	0.90	248
*TECH*	8.20	1.57	3.50	11.17	248
*INEQ*	2.56	0.39	1.73	3.80	248
*GOV*	0.29	0.21	0.12	1.38	248
*EDU*	0.02	0.01	0.01	0.04	248
*PGDP*	0.59	0.28	0.22	1.65	248
*DPOP*	0.29	0.11	0.11	0.55	248
*FINA*	1.93	0.81	0.90	5.59	248
*OPEN*	11.88	4.25	3.06	20.26	248

A series of China statistical yearbooks are the primary data sources.

## Empirical results

### Causal steps approach

Following the discussion in the last chapter about how information infrastructure on industrial structure upgrading, information infrastructure may first affect urbanization level and technological innovation, then finally industrial structure upgrading. At the same time, information infrastructure could also first affect urbanization level, then technological innovation, and finally industrial structure upgrading, that is, there will be an interaction between two mediators. To verify the existence of those pathways, we analyze the impact pathways of information infrastructure on industrial structure upgrading through the mediating effect model proposed by Baron et al.^41^ and the bootstrap test multi-step mediating methods.

Table 3 reports the results of mediating effect models. Column (1) is the benchmark regression results without the two mediating variables of the urbanization process and the technological innovation. Column (2) is the regression results of the relationship between the mediator urbanization process and core independent variable information infrastructure. Column (3) is the regression result of the explained variable industrial structure upgrading, the mediating variable urbanization process, and the core explanatory variable information infrastructure. Among those, column (1) shows that the impact of information infrastructure on industrial structure upgrading is significantly positive; Column (2) shows that the impact of information infrastructure on the urbanization process is significantly positive; Column (3) shows that when information infrastructure and urbanization process are put into regression at the same time, the impact of urbanization process on industrial structure upgrading is significantly positive, and the impact of information infrastructure on industrial structure upgrading is still significantly positive. From those three columns, we conclude that information infrastructure first affects the urbanization process, and then affects industrial structure upgrading. Hypothesis H2 stands, and the urbanization process is a partial mediator.

**Table 3:** Test results of stepwise regression intermediary effect.

	(1)	(2)	(3)	(4)	(5)	(6)	(7)
	*AIS*	*UP*	*AIS*	*TECH*	*TECH*	*AIS*	*AIS*
*UP*			0.593**		7.399***		0.308
			(0.03)		(0.00)		(0.16)
*TECH*						0.047***	0.039**
						(0.00)	(0.01)
*INFOR*	0.110**	0.052**	0.080*	1.300***	0.918***	0.049	0.044
	(0.03)	(0.02)	(0.05)	(0.00)	(0.00)	(0.18)	(0.20)
*INEQ*	− 0.016	− 0.056***	0.018	− 0.504***	− 0.089	0.008	0.021
	(0.36)	(0.00)	(0.34)	(0.00)	(0.61)	(0.65)	(0.18)
*GOV*	0.005	0.116	− 0.064	3.047***	2.191***	− 0.139	− 0.148
	(0.97)	(0.14)	(0.69)	(0.00)	(0.00)	(0.42)	(0.39)
*EDU*	7.018***	5.459***	3.780	4.124	− 36.264*	6.824***	5.180**
	(0.00)	(0.00)	(0.12)	(0.83)	(0.08)	(0.00)	(0.03)
*PGDP*	0.090**	0.023	0.076**	0.761***	0.588*	0.054	0.053
	(0.02)	(0.25)	(0.02)	(0.01)	(0.08)	(0.16)	(0.11)
*DPOP*	0.056	− 0.030	0.074	− 0.470	− 0.245	0.078	0.084
	(0.56)	(0.46)	(0.38)	(0.34)	(0.50)	(0.38)	(0.32)
*FINA*	0.023	0.005	0.020	0.265**	0.225*	0.010	0.011
	(0.17)	(0.43)	(0.21)	(0.04)	(0.07)	(0.54)	(0.50)
*OPEN*	− 0.004***	− 0.003***	− 0.003***	− 0.019***	0.000	− 0.003***	− 0.003***
	(0.00)	(0.00)	(0.01)	(0.00)	(0.95)	(0.00)	(0.00)
*Constant*	2.212***	0.601***	1.855***	7.708***	3.260**	1.848***	1.730***
	(0.00)	(0.00)	(0.00)	(0.00)	(0.02)	(0.00)	(0.00)
*Observations*	248	248	248	248	248	248	248
*R-squared*	0.715	0.845	0.636	0.685	0.751	0.754	0.759

Robust p-values are shown in parentheses. ***, ** and * represent significant coefficients at 0.01, 0.5, and 0.1 significance level respectively.

Column (4) is the regression results between another mediating variable technological innovation and the core explanatory variable information infrastructure. In this case, the coefficient of information infrastructure is significantly positive, indicating that information infrastructure will positively promote technological innovation. Column (5) is the regression results of both mediating variable technological innovation, the urbanization process, and the core explanatory variable information infrastructure. The coefficients of the urbanization process and information infrastructure have passed the significance level test of 1%, and the direction is positive, indicating that the urbanization process will promote technological innovation. Hypothesis H4 holds, that is, column (4), column (2), and column (5) show that information infrastructure first affects the urbanization process, then affects technological innovation, in which the urbanization process plays mediating effect.

Column (6) shows the results when regressing the explanatory variable industrial structure upgrading on mediating variable technological innovation and the core explanatory variable information infrastructure simultaneously. The results show that the impact of technological innovation on industrial structure upgrading is significantly positive, and the impact of information infrastructure on industrial structure upgrading is no longer significant. From columns (1), (4), and (6) we find that information infrastructure first affects technological innovation, then industrial structure upgrading. Hypothesis H3 holds that technological innovation is a complete mediator.

Column (7) depicts a model where industrial structure upgrading is regressed on both mediating variables urbanization process, technological innovation, and the core explanatory variable information infrastructure at the same time. The results show that the impact of technological innovation on industrial structure upgrading is significantly positive, while the impact of the urbanization process on industrial structure upgrading is no longer significant. The comparison of columns (4), (5), and (6), shows that the urbanization process first affects technological innovation, then industrial structure upgrading. Hypothesis H5 holds technological innovation plays a complete mediating effect.

### Bootstrap method

To understand the multiple impact pathways of information infrastructure more accurately on industrial structure upgrading, we also utilize a nonparametric percentile bootstrap method to further test the estimated coefficients and pathways we concluded from the causal steps approach. The results are shown in Table 4. Specifically, in the pathway of “information infrastructure → urbanization → industrial structure upgrading”, the 95% confidence interval from BootLLCI to BootULCI is [0.0608, 0.1197], that is, we have a significant mediating effect from the urbanization level and the effect size is 0.090. Also, the significance test indicates that urbanization has a partial mediating effect. Similarly, technological innovation plays a mediating role in the process of “information infrastructure → technological innovation → industrial structure upgrading”. Notably, urbanization is found to play a partial mediating role in the pathways of “information infrastructure → urbanization → technological innovation”, while technological innovation plays a partial mediating role in the pathways of “urbanization → technological innovation → industrial structure upgrading”. At the same time, the intermediary role of technological innovation (0.119) in the process of information infrastructure affecting industrial structure upgrading is stronger than that of urbanization (0.090).

**Table 4:** Bootstrap test results of mediating effect.

		INFOR → UP → AIS	INFOR → TECH → AIS	INFOR → UP → TECH	UP → TECH → AIS
Ind_eff	Coeffi (Bias)	0.090*** (0.00)	0.119*** (0.00)	0.868*** (0.00)	0.070*** (0.00)
	BC(95% conf. interval)	[0.0608, 0.1197]	[0.0664, 0.1863]	[0.3489, 1.4299]	[0.0316,0.1145]
Dir_eff	Coeffi (Bias)	0.095*** (0.00)	0.065** (0.05)	6.283*** (0.00)	0.398*** (0.00)
	BC(95% conf. interval)	[0.0537, 0.1372]	[0.0059, 0.1293]	[4.9064, 8.0475]	[0.3121, 0.4961]

## Conclusions and policy implications

Using the time series of 31 provinces (excluding Hong Kong, Macao, and Taiwan) in China from 2013 to 2020, we discuss the mediating effects and pathways in the process of information infrastructure impacting industrial structure upgrading. The results show that information infrastructure affects industrial structure upgrading through multiple channels. In addition to the direct impact on industrial structure upgrading, information infrastructure can also play indirect roles through multi-step mediating effects with urbanization and technological innovation as mediating variables. The first pathway is that information infrastructure promotes industrial structure upgrading by promoting urbanization level. The second pathway is information infrastructure promotes industrial structure upgrading by promoting technological innovation. An additional pathway is information infrastructure first promotes technological innovation by promoting urbanization, and eventually promoting industrial structure upgrading. In addition, the intermediary effect of technological innovation is stronger than urbanization.

The findings of this paper further confirm that information infrastructure can promote industrial structure upgrading^6,13–15^. At the same time, based on existing research finding that information infrastructure has promoted urbanization^29,30^ and technological innovation^56–58^, this paper integrates urbanization and technological innovation into the analysis framework of the impact of information infrastructure on industrial structure upgrading. All in all, this study examines the mediating effects and pathways of how information infrastructure impacts industrial structure upgrading and enriches the literature by introducing the interaction mechanism between information infrastructure and industrial structure upgrading. In response to the above conclusions and the fact that China's information infrastructure construction is still at a preliminary stage^43^, we put forward the following policy recommendations: (1) It is necessary to further accelerate the pace of information infrastructure construction. At the same time, enterprises should be encouraged to apply information infrastructure independently to promote the transformation and industrial structure upgrading. (2) Information infrastructures that can promote urbanization and technological innovation should be identified, to accelerate the transformation and upgrading of the industrial structure by adjusting the intermediary path. (3) The layout and construction of information infrastructure should be combined with the development characteristics of specific regions, such as the relationship between urbanization and ecological environment should be comprehensively considered in Kashgar^59^. (4) We should try our best to narrow the gap in regional information infrastructure development and cooperate to realize complementary advantages and mutual benefits.

In addition, there are still some limitations in this work. First, although this study verifies the intermediary role of urbanization and technological innovation in the process of information infrastructure affecting industrial structure upgrading, it is found that there are still other paths to be further explored. Second, our study is also constrained by the availability of a dataset, and so the scientific evaluation of information infrastructure in future research needs to be further strengthened. At the same time, only using Chinese relevant data to be analyzed, and the conclusions drawn need to be further verified internationally.

## Data Availability

The datasets used and/or analysed during the current study available from the corresponding author on reasonable request.
